# Isolated Avulsion Fracture of Patellar Attachment of Medial Patellotibial and Medial Patellomeniscal Ligaments in the Presence of Trochlear Dysplasia: An Indication for Acute Surgical Repair

**DOI:** 10.7759/cureus.6205

**Published:** 2019-11-20

**Authors:** Panagiotis V Samelis, Eftychios Papagrigorakis, Andreas Mavrogenis, Olga Savvidou, Panagiotis Koulouvaris

**Affiliations:** 1 Orthopaedics, Children’s General Hospital Panagiotis & Aglaia Kyriakou, Athens, GRC; 2 Orthopaedics, KAT Trauma Hospital, Athens, GRC; 3 Orthopaedics, School of Medicine National and Kapodistrian University of Athens, Athens, GRC; 4 Orthopaedics, Attikon University Hospital, Athens, GRC; 5 Orthopaedics, Attikon University Hospital, Athens, GRC

**Keywords:** acute, patella, dislocation, mptl, mpml, isolated, avulsion, triangle, repair

## Abstract

We present the case of a 13-year-old female athlete with acute traumatic lateral patellar dislocation. Patella reduced spontaneously with knee extension. Clinical examination revealed pain and tenderness at the middle/inferior part of the medial patellar border. An MRI showed an avulsion fracture at the middle/inferior part of the medial patellar border along with type C dysplastic trochlea and medial femoral condyle hypoplasia. On examination under anesthesia, the patella was stable in extension, indicating intact medial* *patellofemoral ligament (MPFL), but dislocated beyond 30 degrees of knee flexion. Surgical repair of the common patellar attachment of medial patellotibial ligament (MPTL) and medial patellomeniscal ligament (MPML) by means of strong nonabsorbable transosseous sutures was performed, in order to allow healing of MPTL/MPML with the patella centered on the trochlear groove. The follow-up was uncomplicated. It is concluded that isolated acute traumatic insufficiency of MPTL/MPML with coexisting trochlear dysplasia, even with intact MPFL, is an indication for acute surgical MPTL/MPML repair in order to compensate for the inherent osseous instability of the patellofemoral joint.

## Introduction

Medial patellotibial ligament (MPTL) and medial patellomeniscal ligament (MPML) are well-described distal medial passive restraints of the patella. Along with the trochlea, they stabilize the patella beyond 30 degrees of knee flexion after the patella enters the trochlear groove [1,2]. The significance of these ligaments is usually underestimated. Current literature supports MPTL reconstruction only as an augmentation procedure in selected cases in order to support medial patellofemoral ligament (MPFL) reconstruction [3]. However, MPTL/MPML rupture is actually a prerequisite for acute patellar dislocation. Furthermore, it may suffice for patellar dislocation even without MPFL rupture [1,2]. In this study, we aimed to find out if there is an indication for isolated MPTL and MPML acute repair.

## Case presentation

A 13-year-old female athlete presented with an acute lateral dislocation of the left patella. Patella reduced spontaneously with knee extension. The patient had had a contralateral patellar dislocation six months prior, which had been treated conservatively.

**Figure 1:**
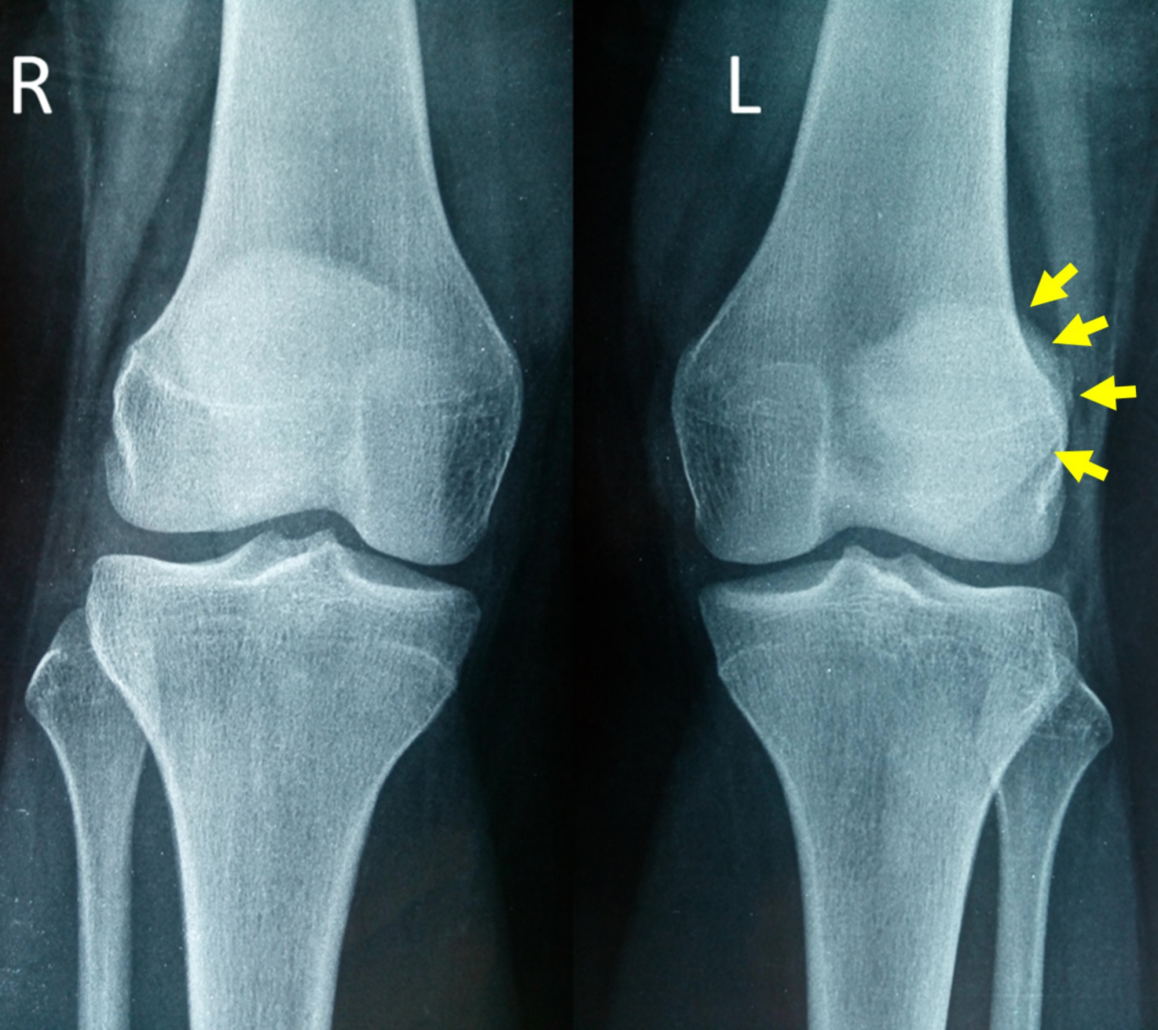
Anteroposterior X-ray of both knees in the standing position on patient admission Arrows indicate lateral subluxation of the left patella

The patient complained of pain and swelling of the left knee. Moderate joint effusion and tenderness at the middle-inferior part of the medial patellar border were detected on clinical examination. No signs of acute MPFL injury (tenderness along the course of the ligament) or other knee pathology (ligament laxity, genu valgum or recurvatum) were manifested. Hip abductors and vastus medialis strength and anatomy were normal. Anteroposterior X-ray of both knees in the standing position showed lateral subluxation of the left patella (Figure 1).

**Figure 2:**
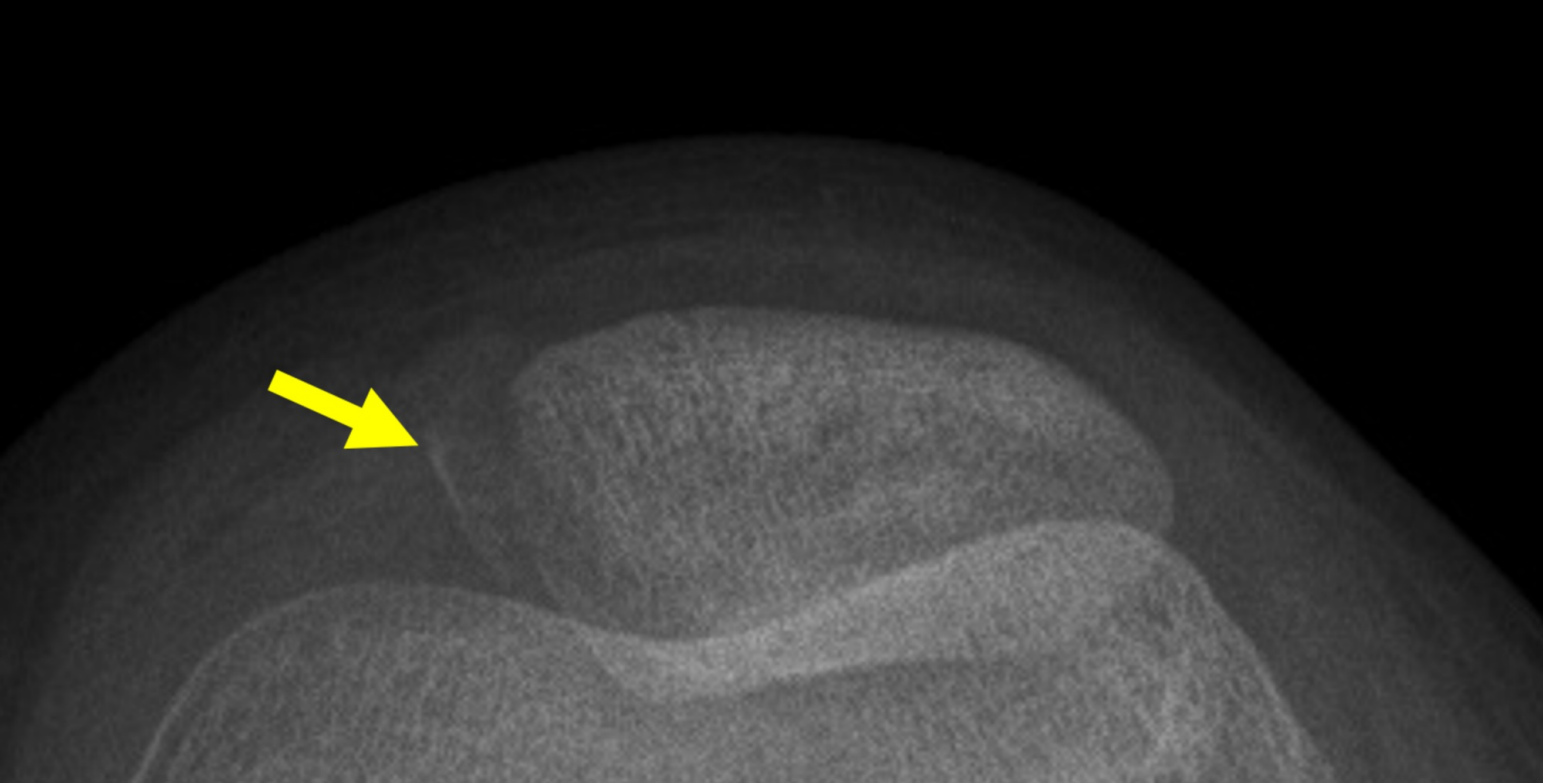
Axial X-ray of the left patella Arrow indicates avulsion fracture of the medial patellar border

The axial X-ray view of the left patella showed an avulsion fracture of the medial patellar border (Figure 2).

Patellar height (patellar tendon length-to-patellar length ratio: 1:16); mechanical (Q angle: 15 degrees) and torsional profile (hip version, posterior cruciate ligament-tibial tubercle distance: <24 mm) of lower limbs were normal.

**Figure 3:**
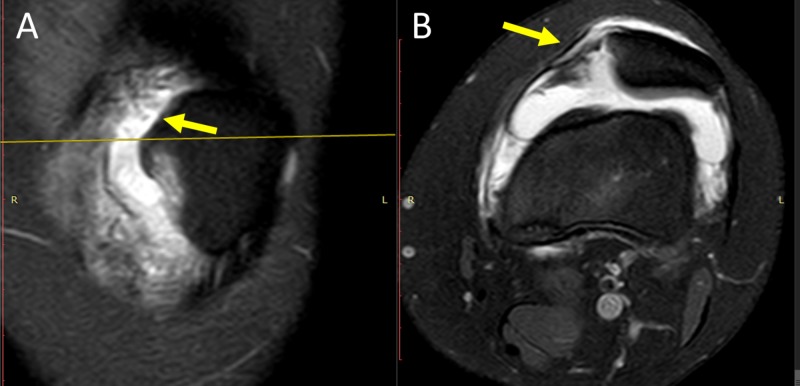
MRI of left Knee: patellar attachment of MPFL A (frontal plane): the arrow indicates the proximal part of the medial patellar border. B (axial plane): the arrow indicates continuous MPFL MRI: magnetic resonance imaging; MPFL: medial patellofemoral ligament

**Figure 4:**
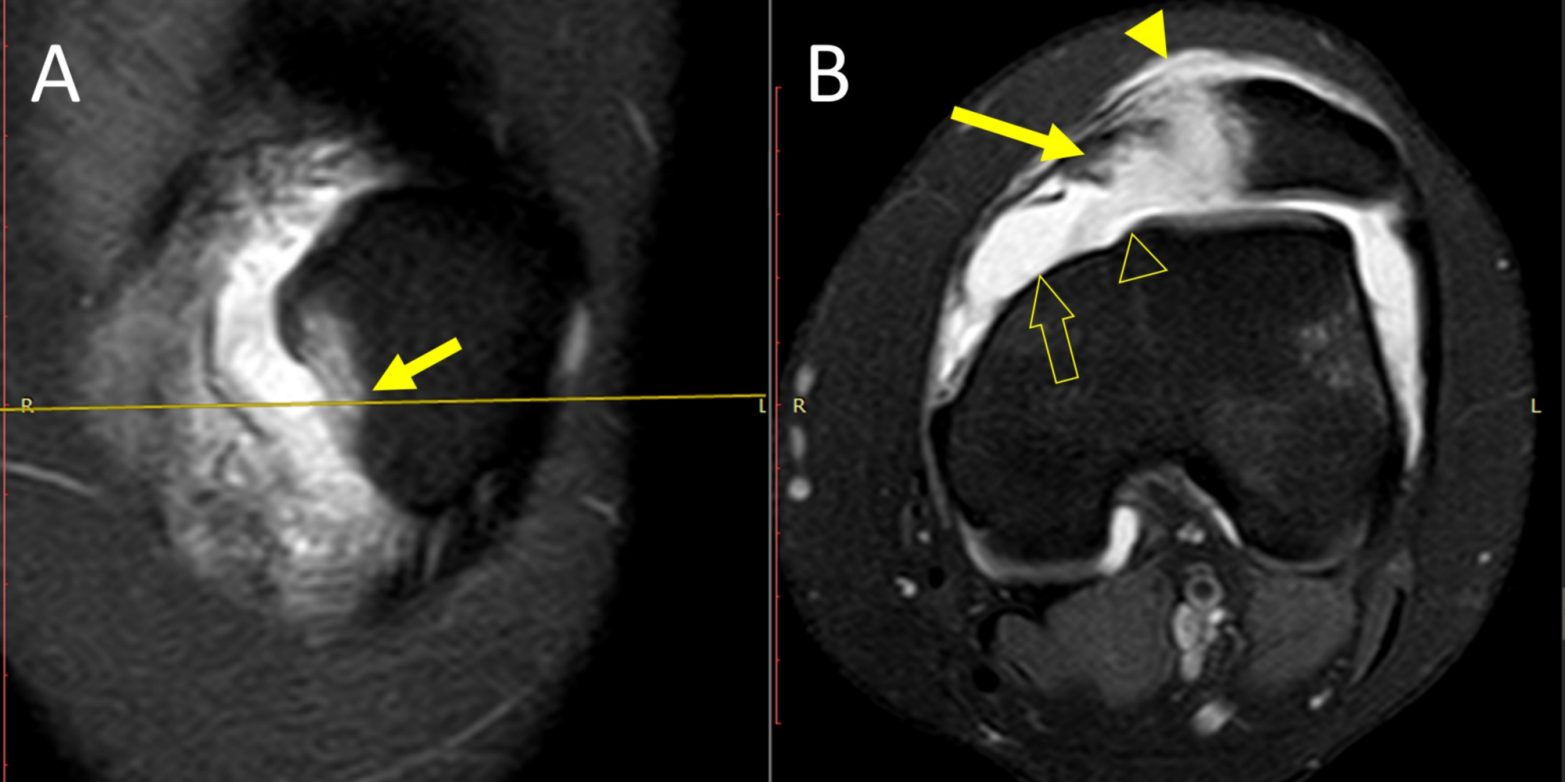
MRI of left knee: patellar attachment of MPTL and MPML A (frontal plane): the arrow indicates the location of avulsion fracture at the distal part of the medial patellar border. B (axial plane, 12 mm distal to the plane of figure 3B): patellar avulsion fracture (solid arrow), complete avulsion of distal medial restraints from the patella (solid arrowhead), hypoplastic medial femoral condyle (arrow outline), flat sulcus (arrowhead outline) MRI: magnetic resonance imaging; MPTL: medial patellotibial ligament; MPML: medial patellomeniscal ligament

The location of the avulsion fracture relative to the patellar attachment of MPFL was determined on left-knee MRI, which showed continuous MPFL attached to the proximalpart of medial patellar border (Figure 3). The avulsion fracture, along with MPTL/MPML attachment, was located at the distal part of medial patellar border. Type C dysplastic trochlea (Dejour's classification) with hypoplastic medial femoral condyle and flat sulcus was evident (Figure 4) [4,5].

Examination under anesthesia (EUA) with the knee extended showed that the patella rested in a subluxed position, but did not dislocate when pushed laterally by the examiner. However, the patella dislocated easily with lateral pressure when the knee was flexed beyond 30 degrees.

Surgical repair of the avulsed medial constraints was decided. MPTL/MPML patellar avulsion was reattached to the middle/inferior medial patellar border by means of strong nonabsorbable transosseous sutures. Intraoperative stability after MPTL/MPML repair was assessed: patella remained centered on the trochlea throughout the full passive range of motion of the knee. The patella did not dislocate beyond 30 degrees of flexion when pushed laterally by the surgeon. The leg was placed in a long limb cast for one month and on a hinged knee brace for another consecutive month. Physical therapy was initiated as soon as pain and swelling subsided. Healing was uncomplicated. The patient returned to her preinjury level of activity at about six months postoperatively.

## Discussion

Several anatomic structures stabilize the patella throughout knee motion. These structures are classified as dynamic (vastus medialis), passive (ligaments: medial and lateral restraints) and static (osseous anatomy: patella, trochlea, tibial tubercle) [1,2,6]. Medial restraints are the main passive stabilizers against lateral patellar dislocation and are further classified as proximal [medial quadriceps tendon femoral ligament (MQTFL), MPFL], and distal (MPTL, MPML) [1,2].

According to biomechanical studies, the proximal medial restraints, collectively known as the medial patellofemoral complex (MPFC), are responsible for about 60% of patellar stability against lateral translation [7,8,9]. However, the proximal medial restraints are most important at the initiation of knee motion, until the patella enters the trochlear groove. After this point, approximately beyond 30 degrees of knee flexion, MPTL/MPML and the trochlea are the main stabilizers against lateral patellar dislocation [1,2,9]. On the other hand, trochlear dysplasia is present in 74% of the patients with acute lateral patellar dislocation [10]. Consequently, the action of MPTL/MPML is crucial when the inherent osseous stability of the trochlear groove is lost, due to underlying trochlear dysplasia [1,7].

**Figure 5:**
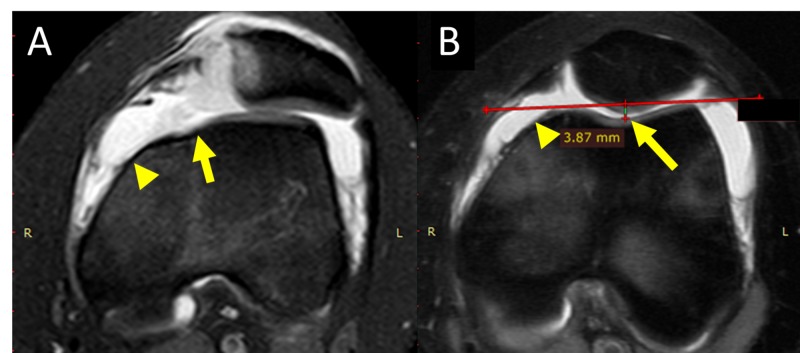
Comparison of the presented case with control. Knee MRI, axial plane 3-cm proximal to knee joint line A: the presented case: type C trochlear dysplasia, shallow trochlear groove (arrow), marked medial femoral condyle hypoplasia (arrowhead). B: control: normal patellofemoral joint, trochlear groove greater than 3 mm (arrow) and normal medial femoral condyle (arrowhead) MRI: magnetic resonance imaging

In the presented case, osseous patellofemoral stability was compromised secondary to type C trochlear dysplasia (Dejour classification), accompanied by marked hypoplasia of the medial femoral condyle and a flat trochlear groove (sulcus angle: >145 degrees; sulcus depth: <3 mm, at 3 cm above knee joint level) [4,5]. MRI of the left knee of a 14-year-old girl, who had an anterior cruciate ligament reconstruction, has been used as a matched control (body weight and height, physical activity) for normal patellofemoral anatomy (Figure 5).

A new concept of patellar instability: the triangular space of patellofemoral dislocation

**Figure 6:**
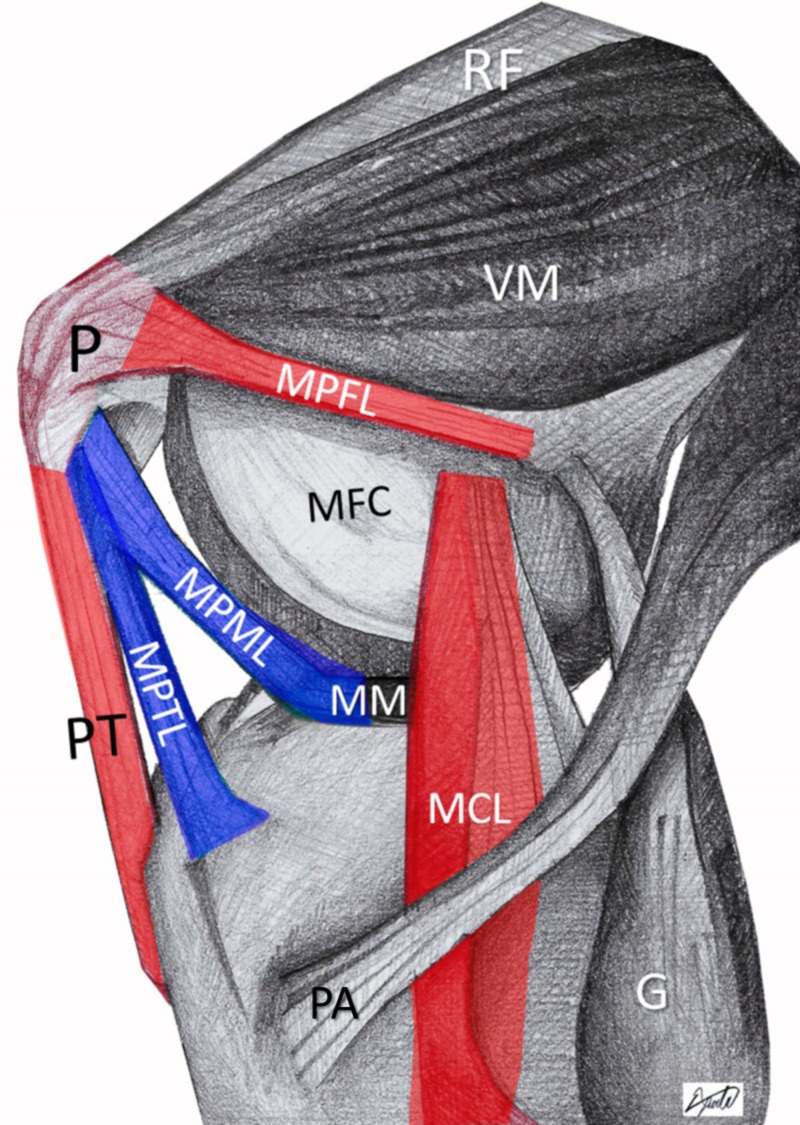
The triangular space of patellofemoral dislocation (red). Patellar dislocation initiates with MPTL/MPML rupture (blue) P: patella; PT: patellar tendon; MFC: medial femoral condyle; MPFL: medial patellofemoral ligament; MPTL: medial patellotibial ligament; MPML: medial patellomeniscal ligament; MM: medial meniscus; MCL: medial collateral ligament; VM: vastus medialis; RF: rectus femoris; PA: pes anserinus; G: gastrocnemius

**Figure 7:**
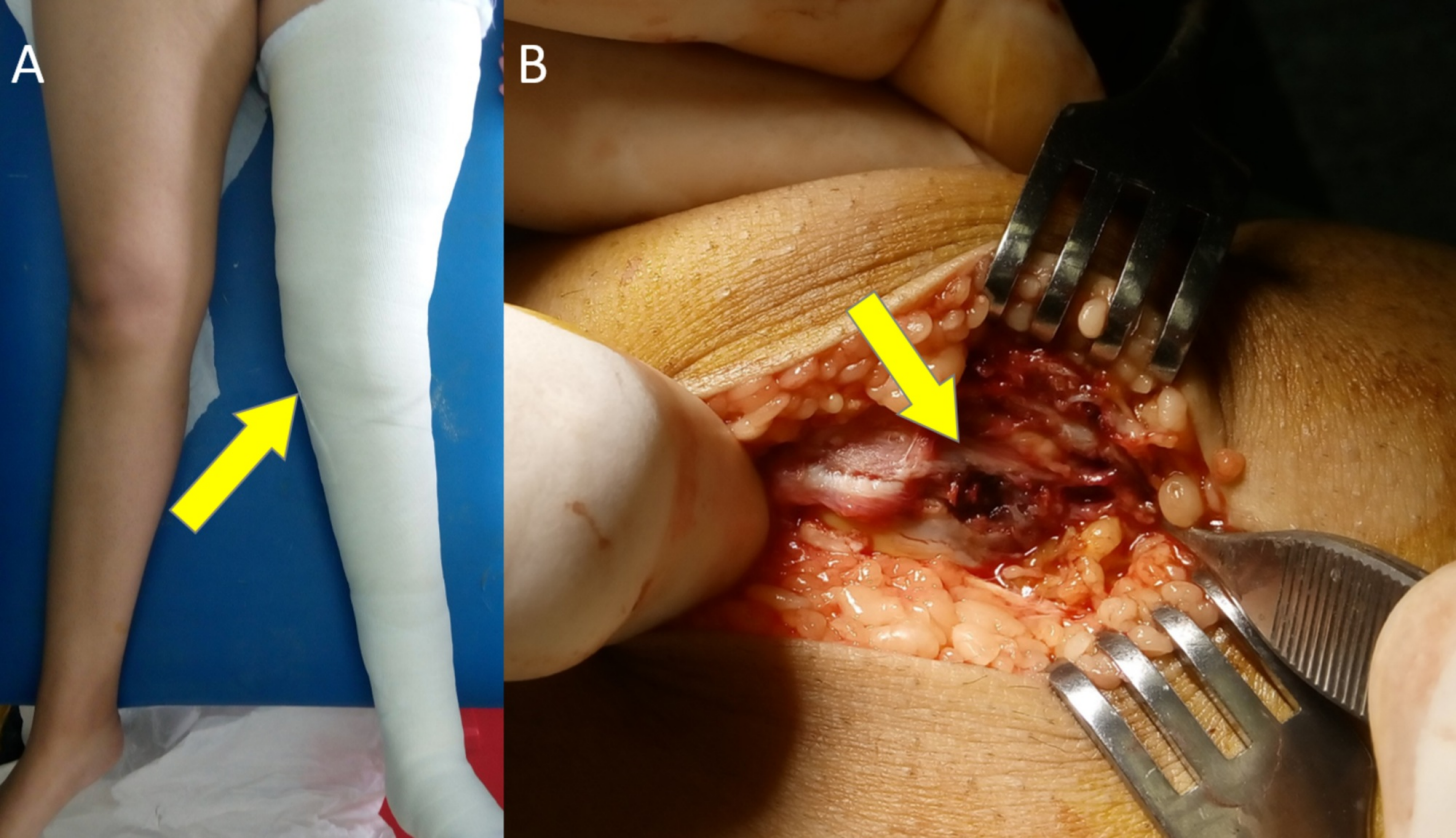
MPTL/MPML patellar avulsion in a 14-year-old girl A.: marked knee valgus, postoperative long limb cast (arrow). B.: intraoperative view of patellar MPTL/MPML avulsion (arrow) MPTL: medial patellotibial ligament; MPML: medial patellomeniscal ligament

In acute traumatic lateral patellar dislocation, it is not only the patella that dislocates. In fact, the femoral condyles “buttonhole” through a triangular space that is bounded by the patella-patellar tendon (P-PT), medial collateral ligament (MCL), and the proximal medial patellar restraints (MQTFL, MPFL) (Figure 6). MPTL and MPML form a barrier that reinforces the surface of this triangle [11,12]. This barrier has to be breached if the patellar dislocation is to occur. However, even after MPTL/MPML injury, the patellar dislocation will not evolve, unless additional comorbidities, that affect patellofemoral stability, are present. Comorbidities, such as MPFL insufficiency (trauma, ligament laxity), patella alta, valgus knee, trochlear dysplasia, etc, predispose to patellar instability by weakening and/or increasing the surface of this triangular space. In the presented case, coexisting trochlear dysplasia sufficed for patellar dislocation after isolated MPTL/MPML trauma. In Figure 7, a similar case of MPTL/MPML patellar avulsion in a 14-year-old girl with marked knee valgus and patella alta is shown.

Patellar dislocation without MPFL rupture?

Current knowledge supports that MPML/MPTL injury may be a prerequisite for the patella to dislocate [1,2,13]. MPTL and MPML are stiffer than MPFL and, therefore, may rupture with less deformation, or even without patellar dislocation and MPFL injury [2,11,13]. In the presented case, avulsion of the common patellar attachment of MPTL/MPML, combined with a dysplastic, shallow trochlea and a hypoplastic medial condyle, resulted in patellar dislocation without concomitant MPFL rupture. After reduction, the patella rested in a subluxed position, secondary to coexisting trochlear dysplasia; however, the patella did not dislocate during EUA with the knee in extension, due to MPFL integrity.

A possible explanation is that the length of MPFL is determined developmentally by the position of the patella with the knee extended (e.g., standing, sleeping). At this point, MPFL assumes its maximal length. In case of trochlear dysplasia with a hypoplastic medial femoral condyle, MPFL is relatively longer than needed when the knee is flexed beyond 30 degrees (patella enters trochlea). Thus, after this point, patellar dislocation may still evolve without simultaneous MPFL injury (rupture, avulsion, mid-substance tear, plastic deformation), provided that MPTL and MPML are ruptured or avulsed.

A clinical sign

Posttraumatic patellar instability beyond 30 degrees of knee flexion with the patella stable in extension indicates isolated acute MPTL/MPML insufficiency.

A surgical indication

Isolated acute MPTL/MPML insufficiency with underlying trochlear dysplasia is an indication for acute surgical repair of MPTL/MPML in order to compensate for the inherent osseous instability of the patellofemoral joint. In the presented case, direct transosseous suturing of the patellar attachments of MPTL/MPML allowed healing of the distal medial patellar restraints with the patella centered on the trochlea. If not repaired, MPTL/MPML would probably heal with the patella subluxed, thus increasing the risk of recurrent patellar instability. Clinically, this would be documented as a positive patellar apprehension at higher degrees of knee flexion [14].

## Conclusions

A wide spectrum of pathologic conditions, at or away from the knee joint, solely or in concert, can lead to patellar instability. The concept of the “triangular space of patellofemoral dislocation” integrates most currently described factors that predispose to lateral patellar instability. Posttraumatic patellar dislocation in flexion with the patella stable in extension may represent a clinical sign indicative of isolatedMPTL/MPML insufficiency without simultaneous MPFL insufficiency. This sign, in the presence of comorbidities that favor patellar dislocation, such as trochlear dysplasia, is an indication for acute surgical repair of MPTL/MPML injury in order to allow healing of the medial restraints with the patella anatomically reduced.
